# Supplementary material for: Benchmarking computational methods for single-cell chromatin data analysis
Source: Genome Biol. 2024 Aug 16;25:225. doi: 10.1186/s13059-024-03356-x (PMC11328424; doi:10.1186/s13059-024-03356-x)
Supplement: Supplementary file 1 — Additional file 1. Supplementary figures S1-21 [file 13059_2024_3356_MOESM1_ESM.pdf]

Fig S1

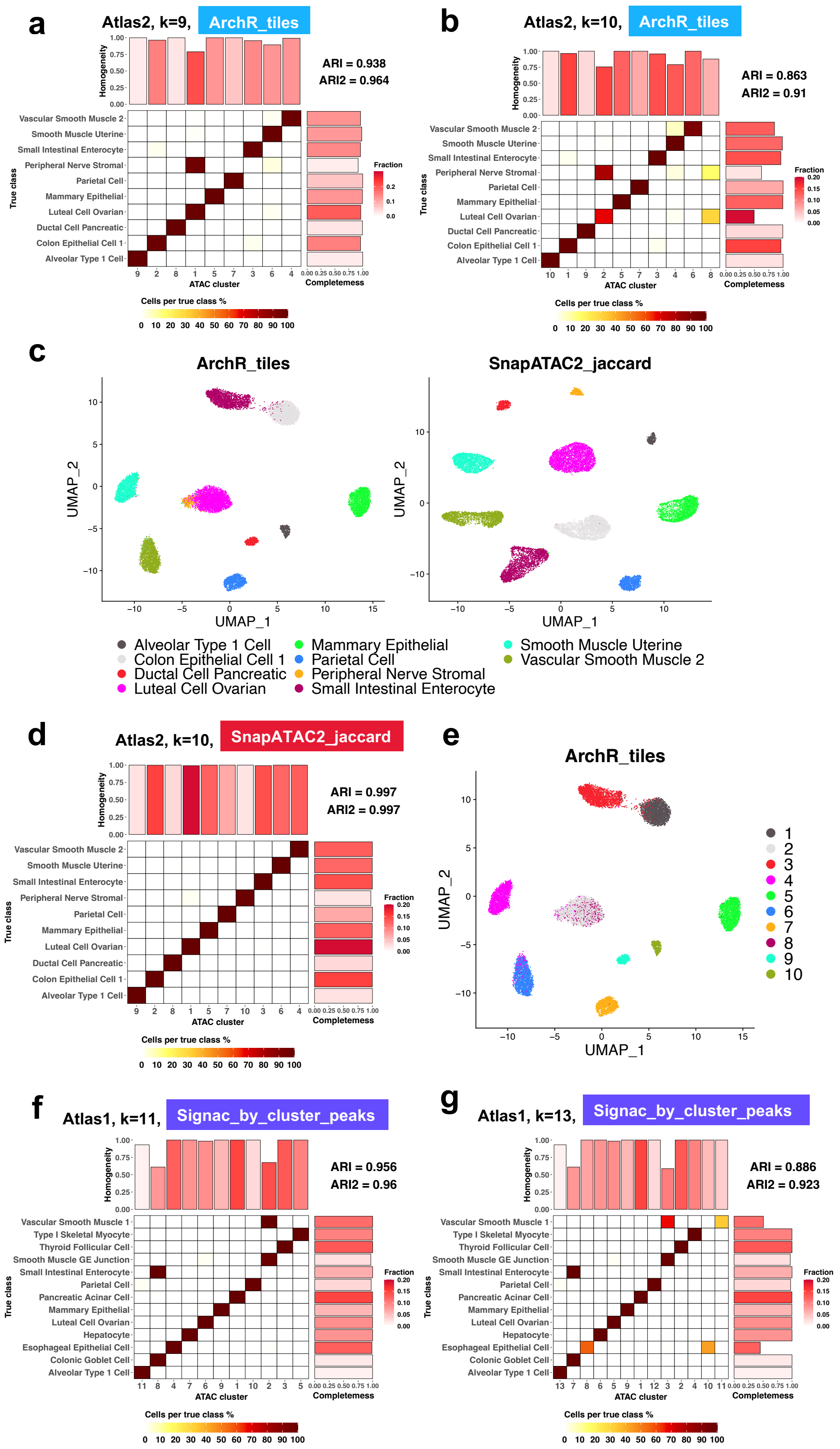
a
Atlas2, k=9, ArchR_tiles
ARI = 0.938
ARI2 = 0.964
b
Atlas2, k=10, ArchR_tiles
ARI = 0.863
ARI2 = 0.91
Homogeneity
True class
ATAC cluster
Completeness
Fraction
Cells per true class %
Vascular Smooth Muscle 2
Smooth Muscle Uterine
Small Intestinal Enterocyte
Peripheral Nerve Stromal
Parietal Cell
Mammary Epithelial
Luteal Cell Ovarian
Ductal Cell Pancreatic
Colon Epithelial Cell 1
Alveolar Type 1 Cell
c
ArchR_tiles
SnapATAC2_jaccard
UMAP_1
UMAP_2
Alveolar Type 1 Cell
Colon Epithelial Cell 1
Ductal Cell Pancreatic
Luteal Cell Ovarian
Mammary Epithelial
Parietal Cell
Peripheral Nerve Stromal
Small Intestinal Enterocyte
Smooth Muscle Uterine
Vascular Smooth Muscle 2
d
Atlas2, k=10, SnapATAC2_jaccard
ARI = 0.997
ARI2 = 0.997
e
ArchR_tiles
f
Atlas1, k=11, Signac_by_cluster_peaks
ARI = 0.956
ARI2 = 0.96
g
Atlas1, k=13, Signac_by_cluster_peaks
ARI = 0.886
ARI2 = 0.923
Vascular Smooth Muscle 1
Type I Skeletal Myocyte
Thyroid Follicular Cell
Smooth Muscle GE Junction
Small Intestinal Enterocyte
Parietal Cell
Pancreatic Acinar Cell
Mammary Epithelial
Luteal Cell Ovarian
Hepatocyte
Esophageal Epithelial Cell
Colonic Goblet Cell
Alveolar Type 1 Cell

Figure S1: **a,b** Clustering solutions by ArchR_tiles on Atlas2, with different numbers of clusters identified. **c** UMAP of Atlas2 generated by ArchR_tiles and SnapATAC2_jaccard, annotated by the true classes. **d** The corresponding clustering solutions by SnapATAC2_jaccard on Atlas2. **e** UMAP annotated by the clustering solutions of ArchR_tiles in **b**. Cluster 8 is the wrongly identified clusters. **f,g** Clustering solutions by Signac_by_cluster_peaks in dataset Atlas1, with different numbers of clusters identified.

Fig S2

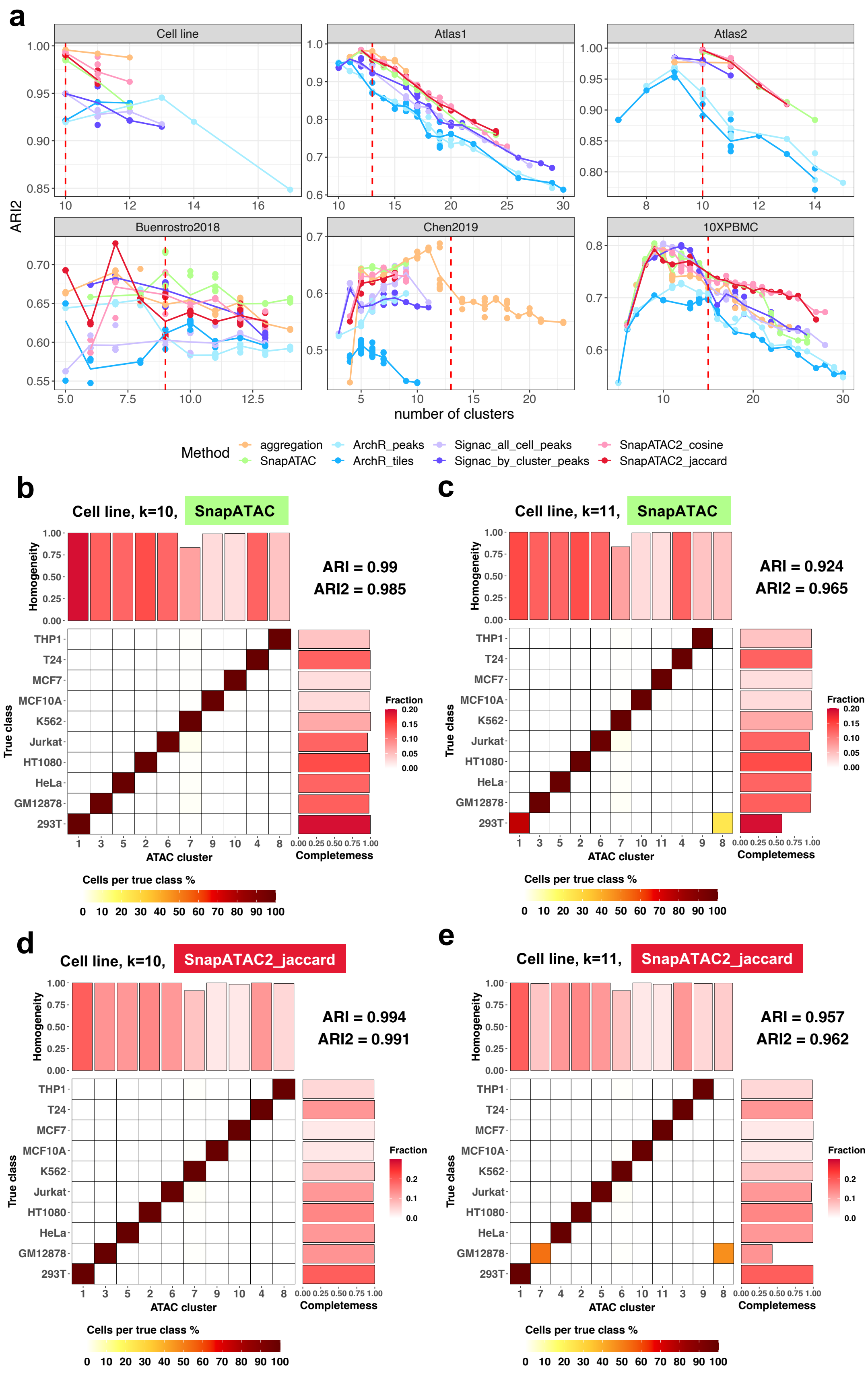
a
Cell line
Atlas1
Atlas2
Buenrostro2018
Chen2019
10XPBMC
ARI2
number of clusters
Method
aggregation
SnapATAC
ArchR_peaks
ArchR_tiles
Signac_all_cell_peaks
Signac_by_cluster_peaks
SnapATAC2_cosine
SnapATAC2_jaccard
b
Cell line, k=10,
SnapATAC
ARI = 0.99
ARI2 = 0.985
c
Cell line, k=11,
SnapATAC
ARI = 0.924
ARI2 = 0.965
d
Cell line, k=10,
SnapATAC2_jaccard
ARI = 0.994
ARI2 = 0.991
e
Cell line, k=11,
SnapATAC2_jaccard
ARI = 0.957
ARI2 = 0.962
Homogeneity
True class
THP1
T24
MCF7
MCF10A
K562
Jurkat
HT1080
HeLa
GM12878
293T
ATAC cluster
Completemess
Fraction
Cells per true class %

Figure S2: **a** ARI2 plotted against various number of clusters; as shown in Figure 3a, each subpanel represents a dataset. Each point represents a clustering solution obtained by varying the resolution parameter and the random seed in Leiden algorithm. The line plot is the average ARI2 at a given number of clusters. **b**-**e** True classes and their fractions of agreement with the predicted clusters. **b**-**c** are SnapATAC on dataset Cell line, and **d**-**e** are SnapATAC2_jaccard on Cell line. The x-axis is the predicted clusters, and the y-axis is the ground truth classes. The colors of tiles indicate the proportion of cells from the corresponding true class (each row sums up to one). A clearer diagonal structure indicates better agreement. ARI and ARI2 are calculated and shown on the top right. The barplot on top shows the value of AV (Methods) and can be interpreted as the homogeneity of the corresponding clusters. The barplot on the right shows the value of AW and represents the completeness of each true class in the prediction. The color of the bars shows the proportion of cells in each cluster/ground truth class. In title, the corresponding datasets, methods, and number of clusters are indicated.

Fig S3

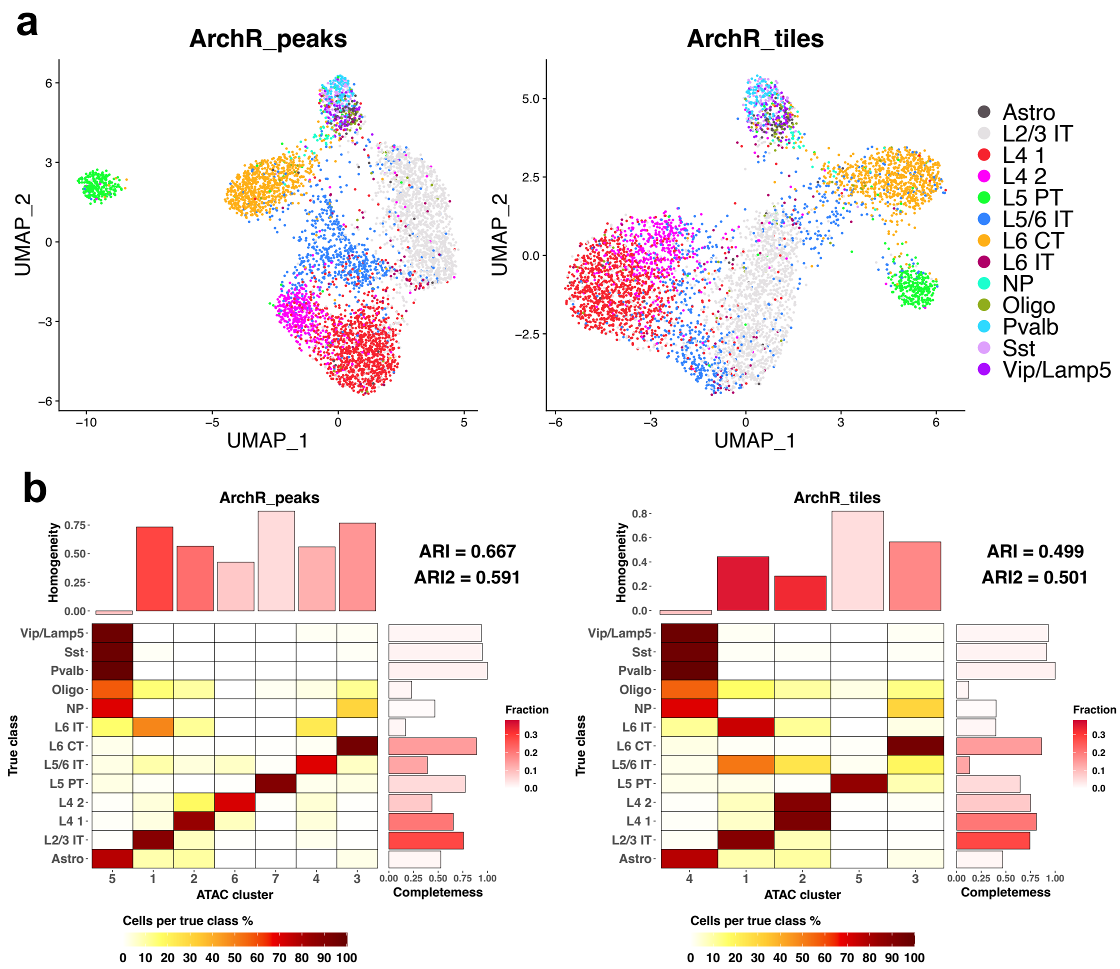

Figure S3: aUMAP of Chen2019 generated by ArchR_peaks and ArchR_tiles. **b** Clustering solutions of the highest ARI by ArchR_peaks and ArchR_tiles on Chen2019.

Fig S4

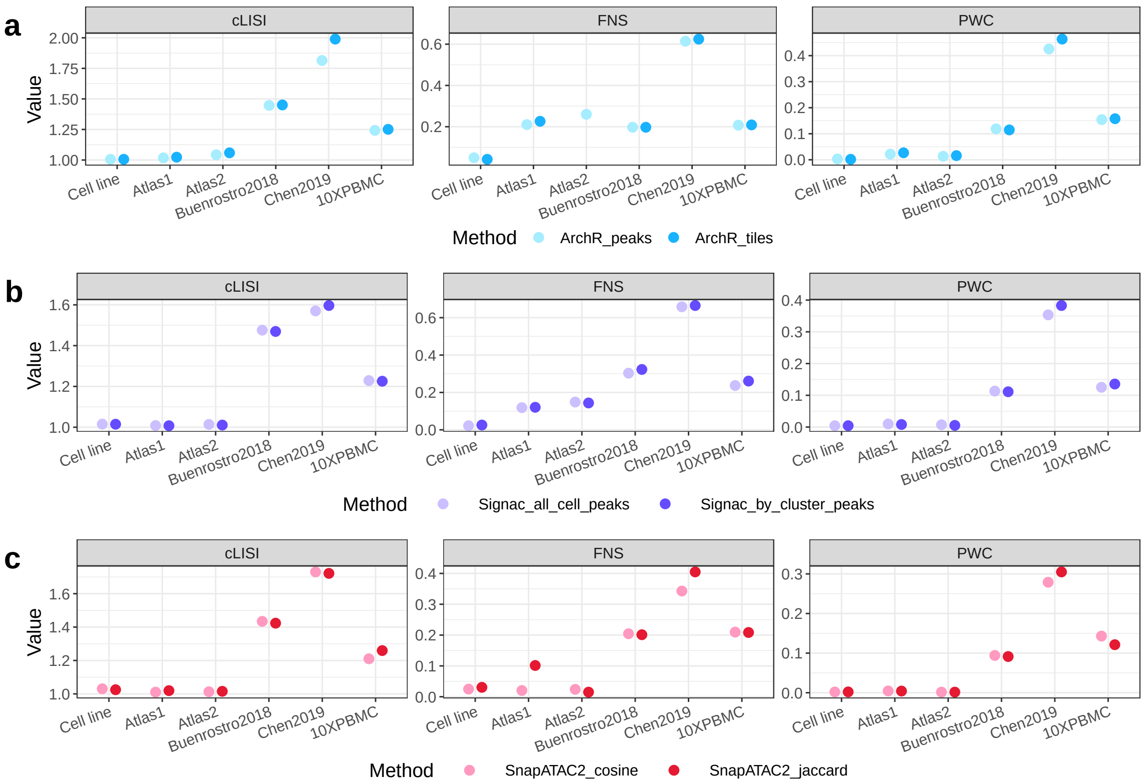

Figure S4: Comparison of the method performance between different data processing choices, measured by the embedding and graph-level metrics. **a** Comparing ArchR_peaks and ArchR_tiles. **b** Comparing Signac_all_cell_peaks and Signac_by_cluster_peaks. **c** Comparing SnapATAC2_cosine and SnapATAC2_jaccard.

Fig S5

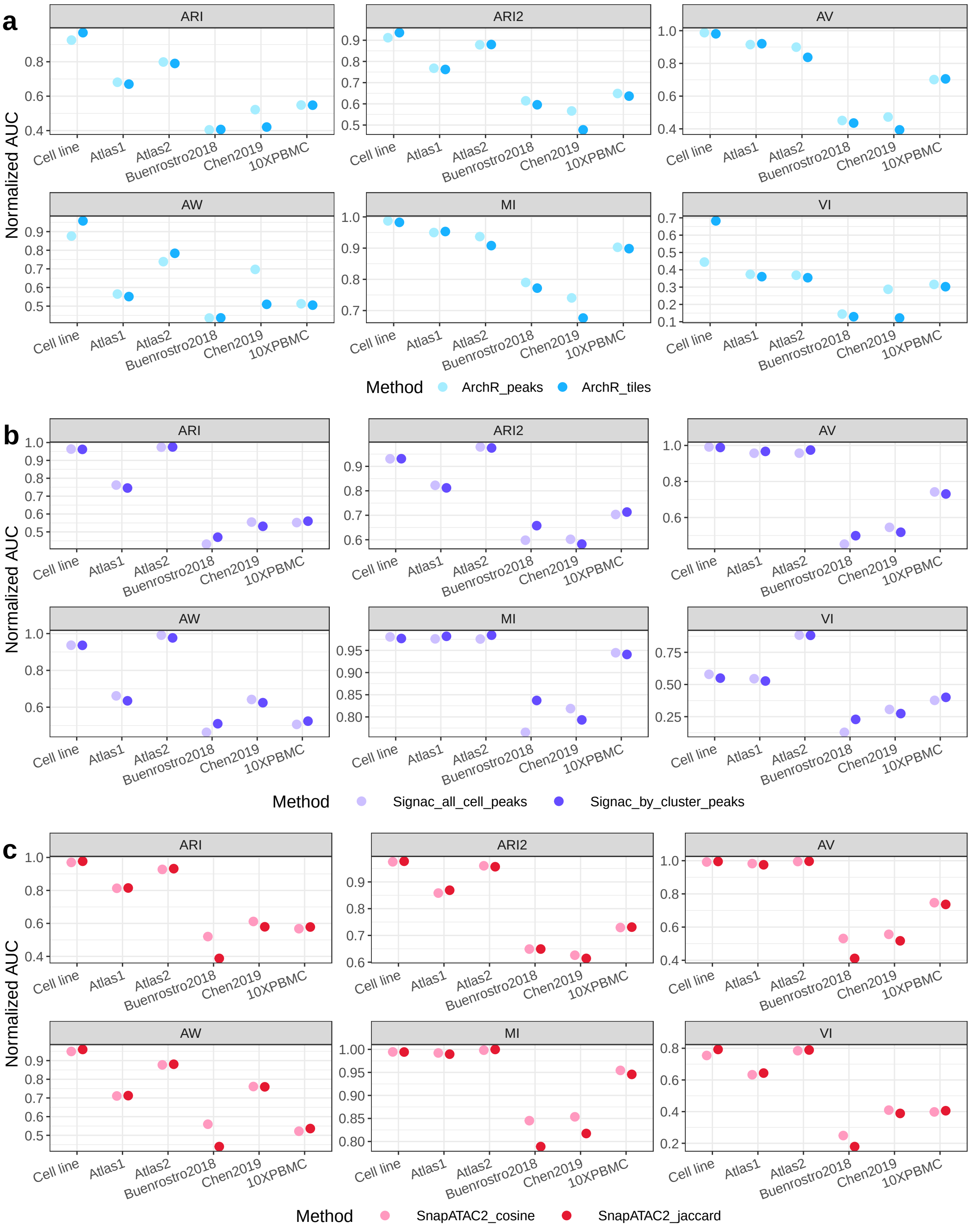

Figure S5: Comparison of the method performance between different data processing choices, measured by the partition-level metrics. **a** Comparing ArchR_peaks and ArchR_tiles. **b** Comparing Signac_all_cell_peaks and Signac_by_cluster_peaks. **c** Comparing SnapATAC2_cosine and SnapATAC2_jaccard.

Fig S6

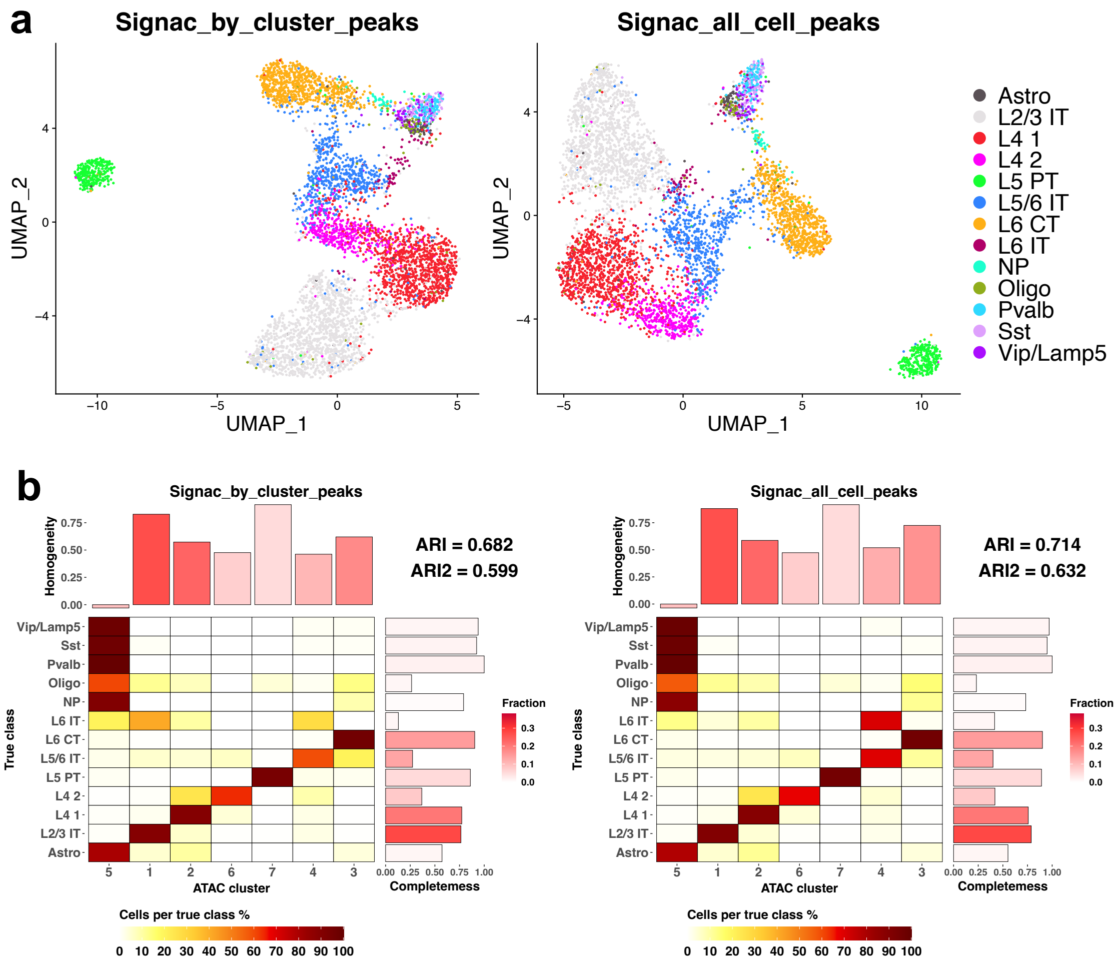

Figure S6: aUMAP of Chen2019 generated by Signac_by_cluster_peaks and Signac_all_cell_peaks. **b** Clustering solutions of the highest ARI by Signac_by_cluster_peaks and Signac_all_cell_peaks on Chen2019.

Fig S7

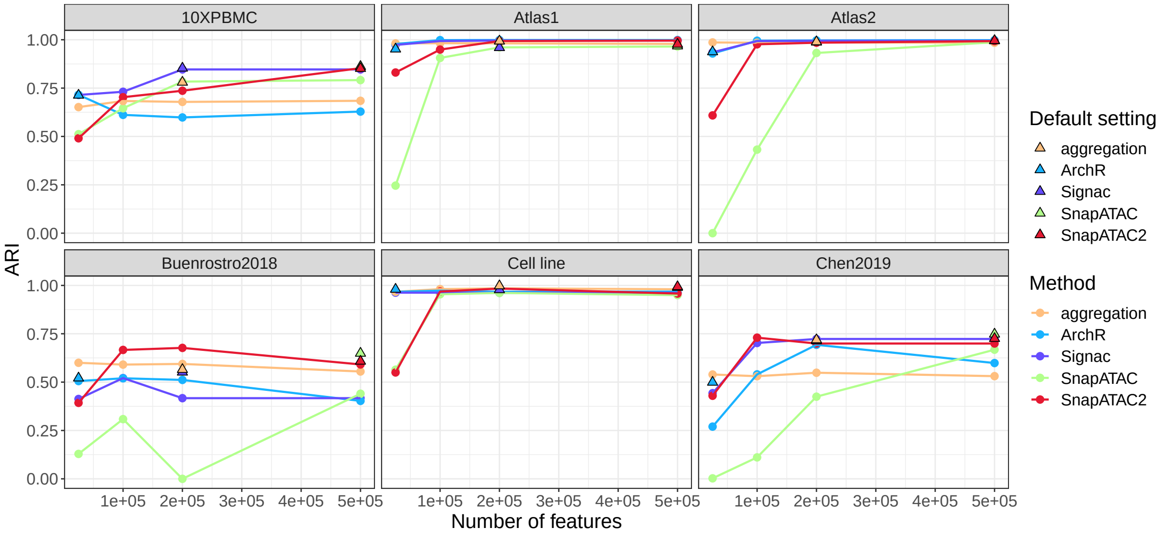

Figure S7: How the ARI changes as the number of features changes. The triangles are showing the ARI of each method using default settings, while the round points are showing the ARI of independent runs with different feature numbers.

Fig S8

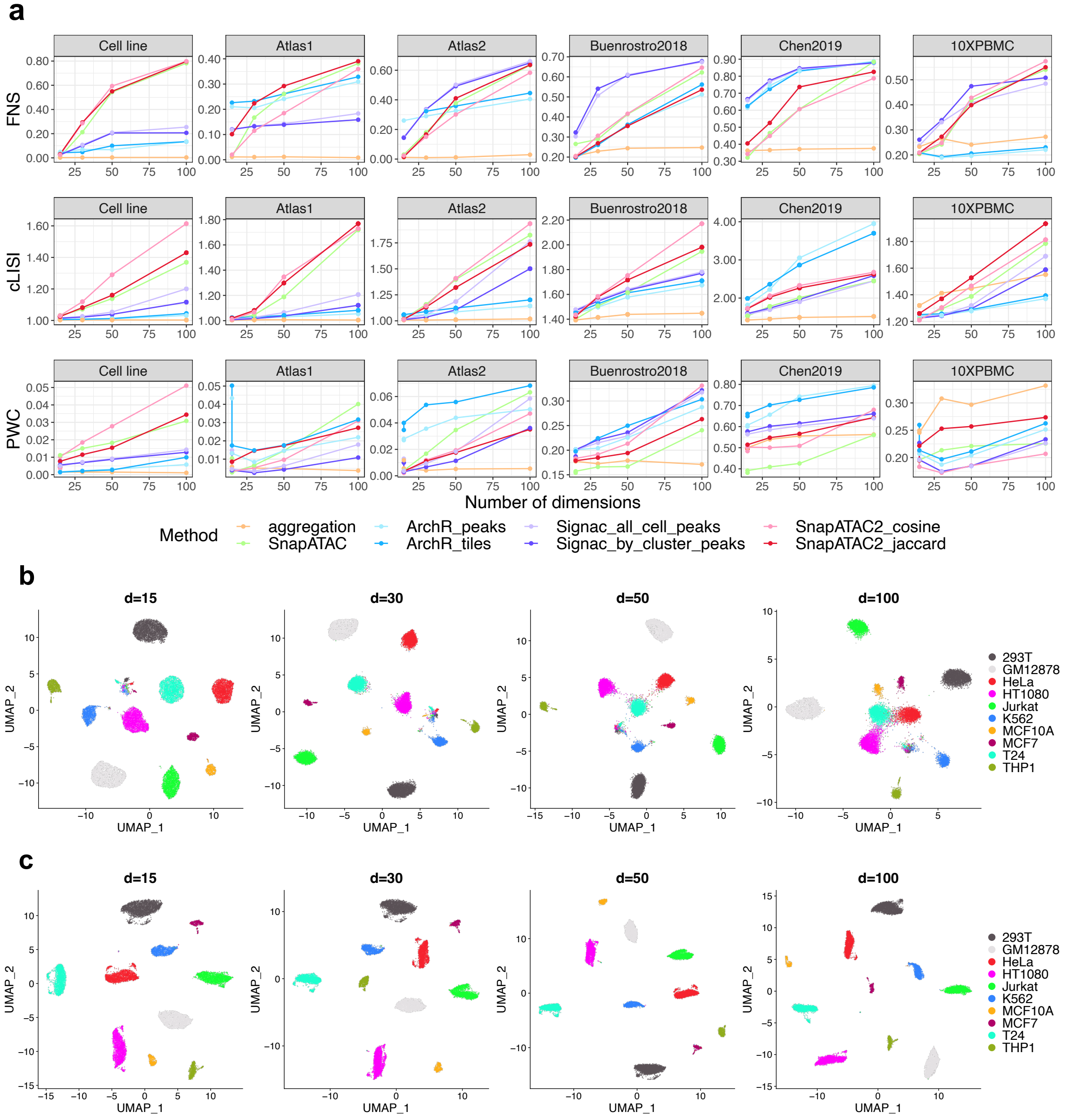

Figure S8: **a** Performance of each method across different choices of the number of latent dimensions, evaluated using embedding-level and graph-level metrics. **b** UMAP of Cell line dataset using SnapATAC2_cosine method. **c** UMAP of Cell line dataset using aggregation method.

Fig S9

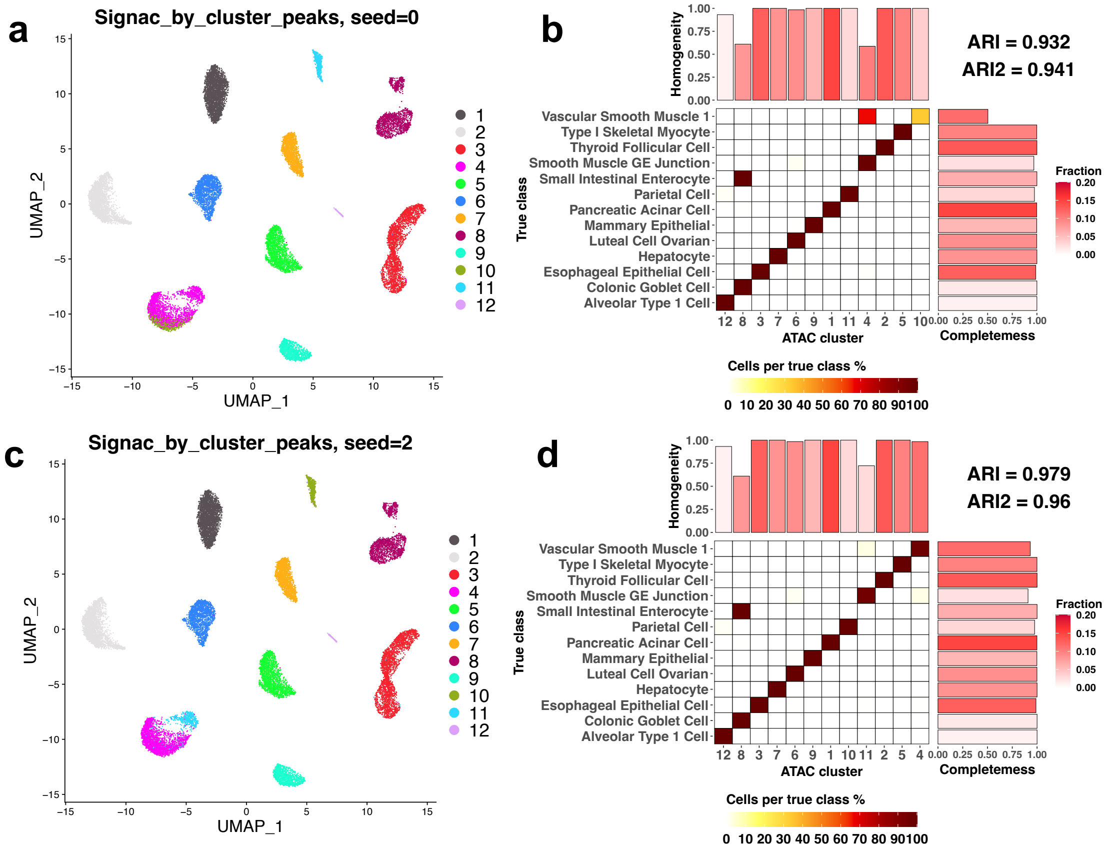

Figure S9: Clustering solutions of Signac_by_cluster_peaks on Atlas1, using random seed 0 (**a**) or random seed 2 (**b**) when running Leiden algorithm.

Fig S10

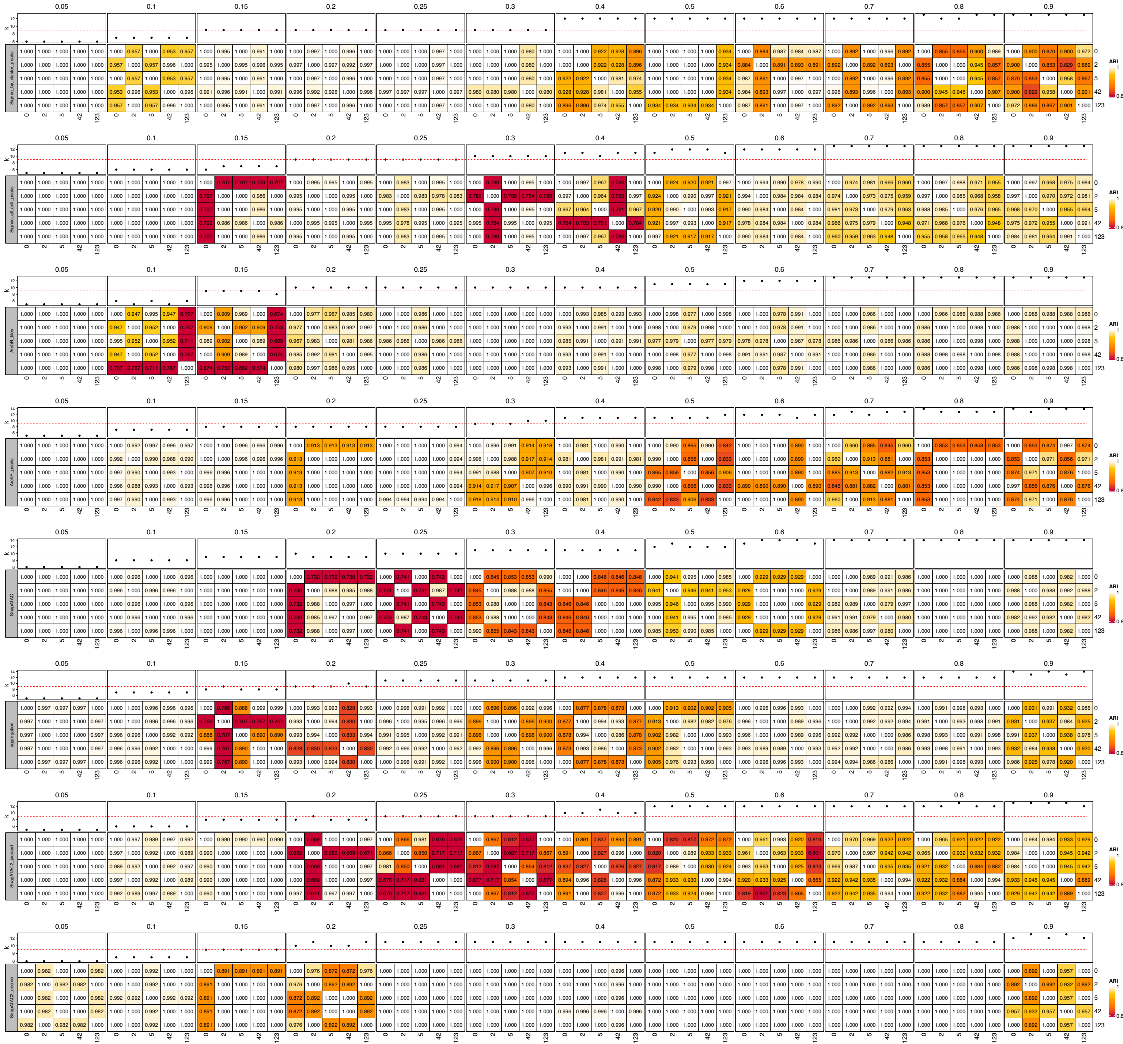

Figure S10: ARI between clustering solutions using the 5 random seeds, in combination with different methods and resolution parameters in dataset Buenrostro2018. The dot plots on top show the number of clusters, and the red horizontal lines are the ground truth cluster numbers.

Fig S11

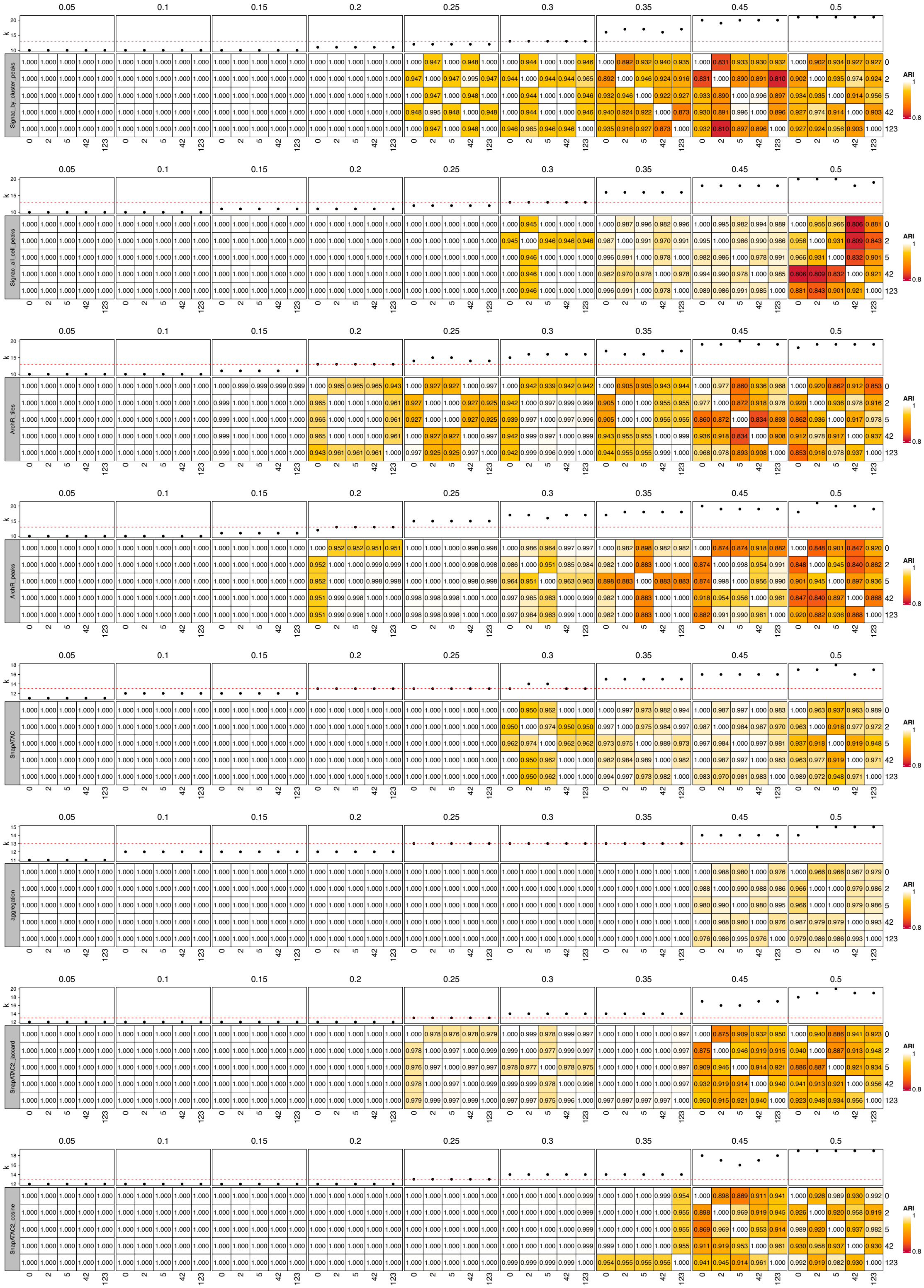

Figure S11: ARI between clustering solutions using the 5 random seeds, in combination with different methods and resolution parameters in dataset Atlas1. The dot plots on top show the number of clusters, and the red horizontal lines are the ground truth cluster numbers.

Fig S12

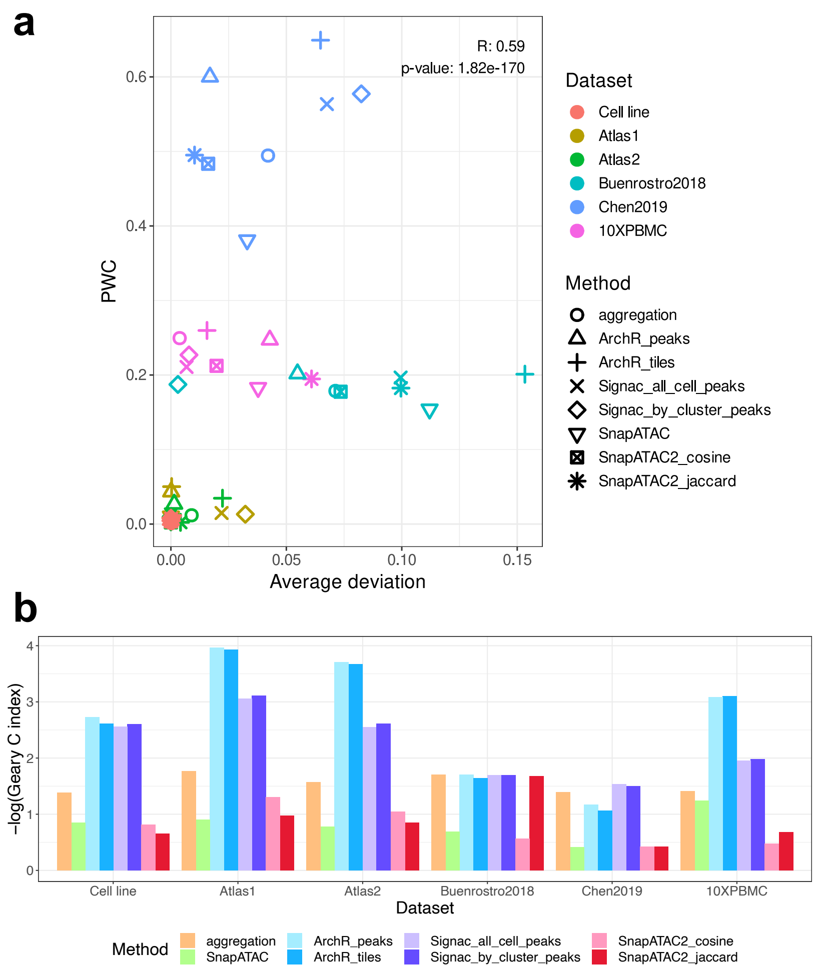

Figure S12: a Related to Figure S10, S11. x-axis is the deviation of ARI from 1, averaged across resolutions and random seeds. y-axis is the averaged PWC score of each dataset and method. **b** Spatial autocorrelation of library sizes measured by Geary's C index.

Fig S13

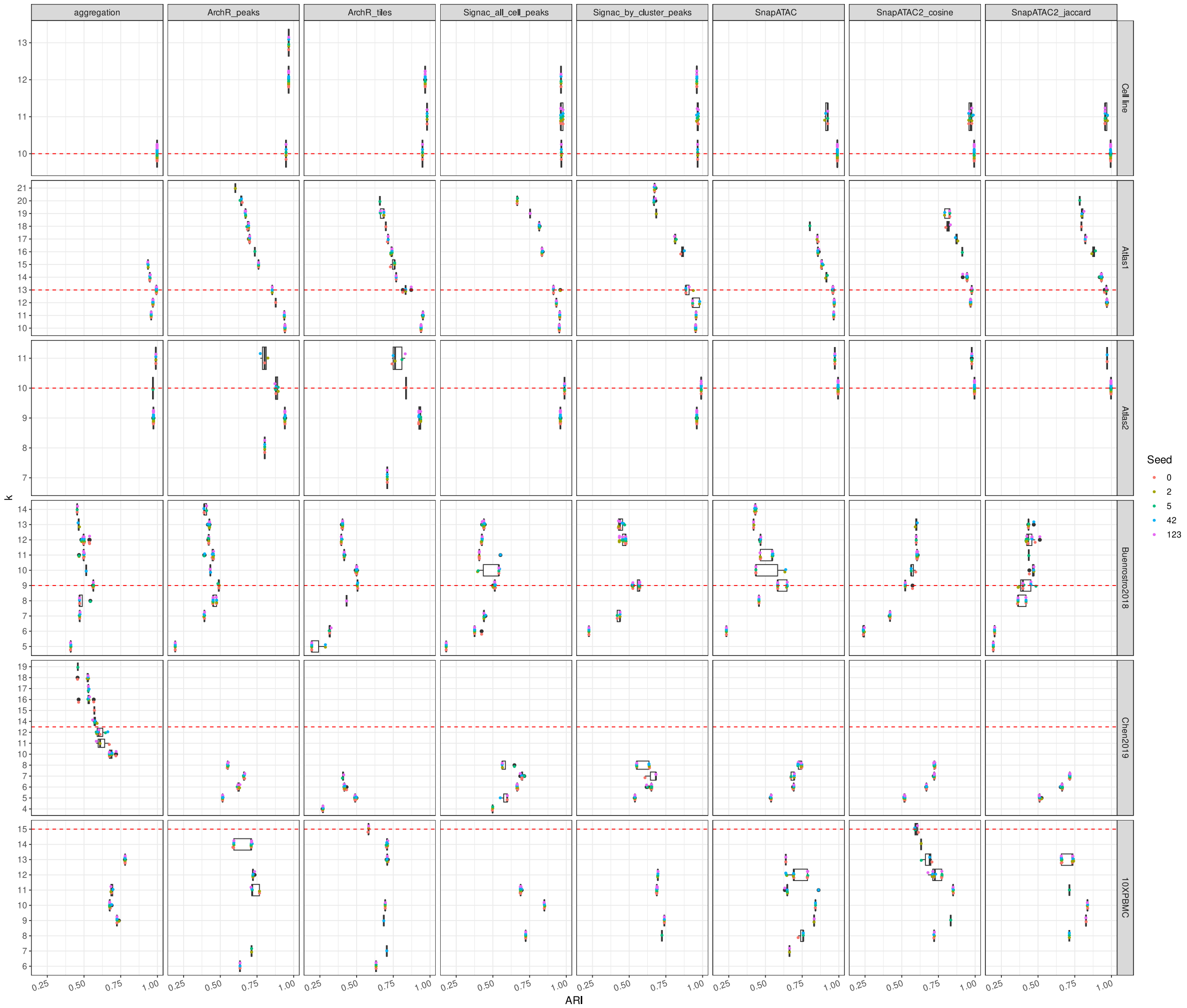

Figure S13: Boxplots of ARI between predicted clusterings and the true cell types, across datasets, methods, and the predicted number of clusters. The red horizontal lines are the ground truth cluster numbers.

Fig S14

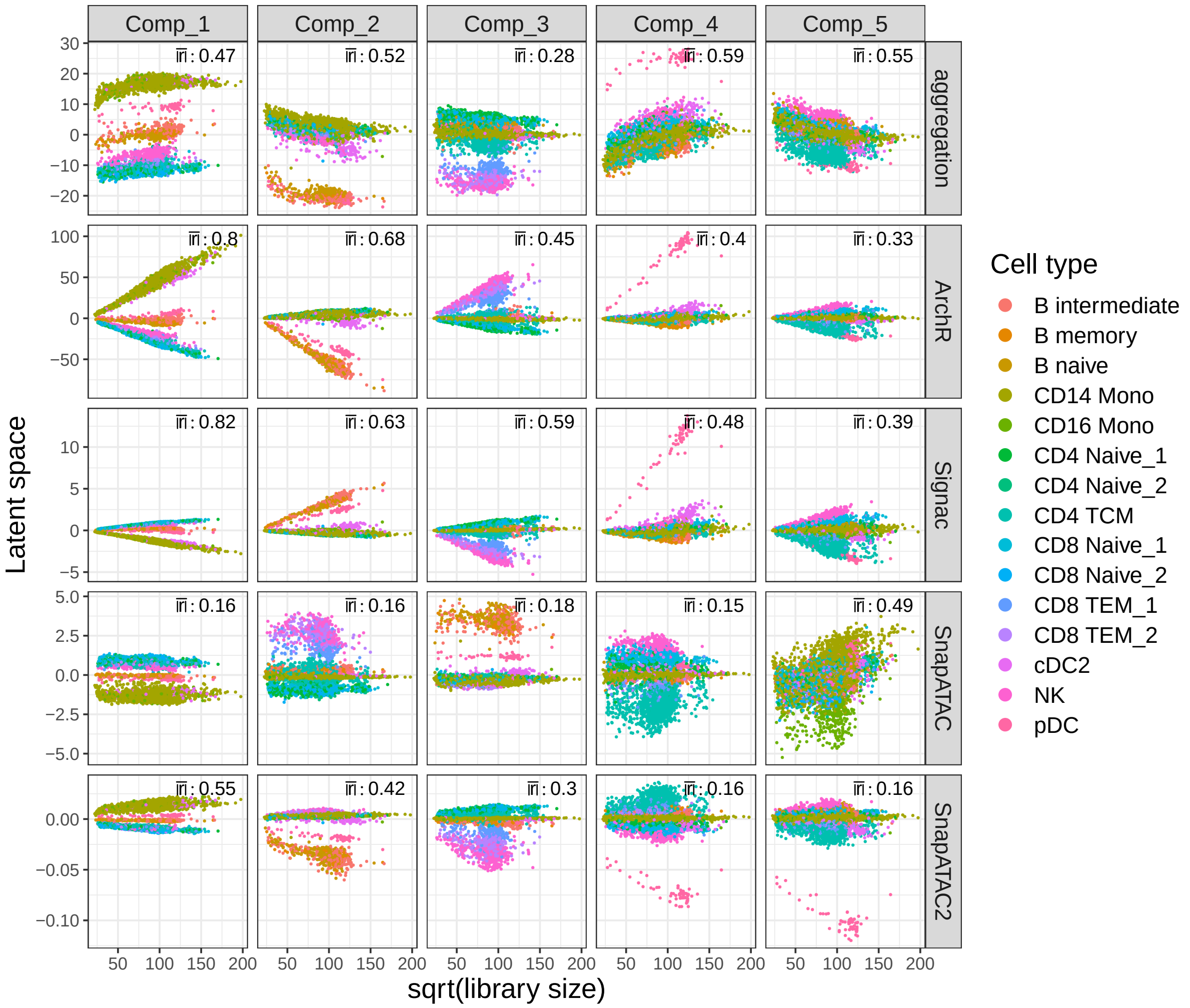

Figure S14: Scatter plots of the latent component value against the square root of fragment counts for dataset 10XPBMC. Colors are cell types, and the absolute Pearson's correlation coefficient between x- and y-axis are calculated and averaged across cell types for each latent components. See Methods for the details about the calculation.

Fig S15

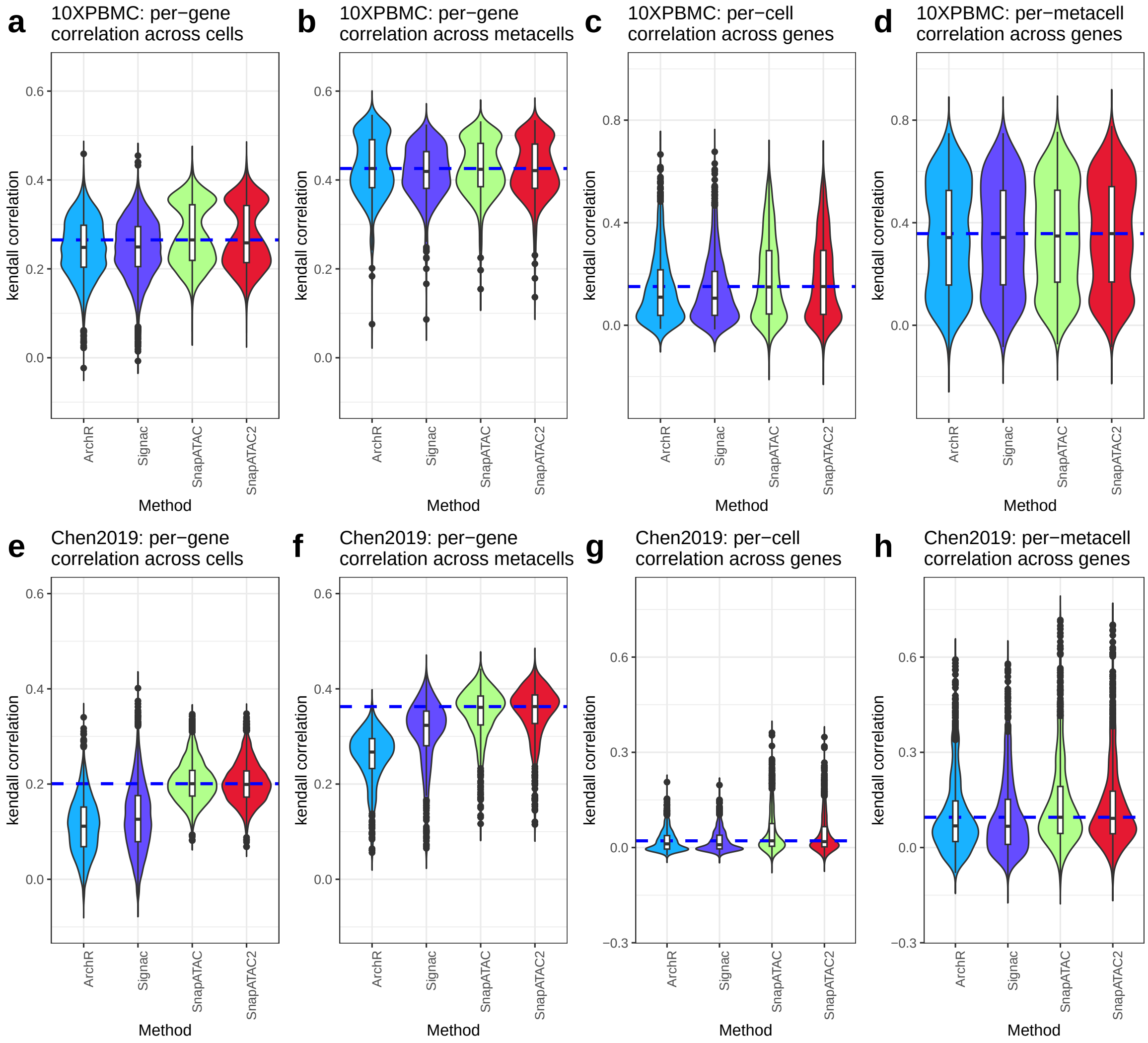

Figure S15: Distribution of Kendall's correlations between the inferred gene activity score and the aligned gene expression. **a**-**d** are data from 10XPBMC dataset, and **e**-**h** are data from Chen2019 dataset. In **a, b, e**, and **f**, the per-gene correlations are calculated for each cell (**a,e**) or each metacell (**b,f**) (500 metacells in total). In **c** and **g**, the per-cell correlations are calculated for each gene. In **d** and **h**, the per-metacell correlations are calculated for each gene. The blue dashed line represents the median value of the best-performing model. Violin plots represent the smoothed density of the distribution of the data.

Fig S16

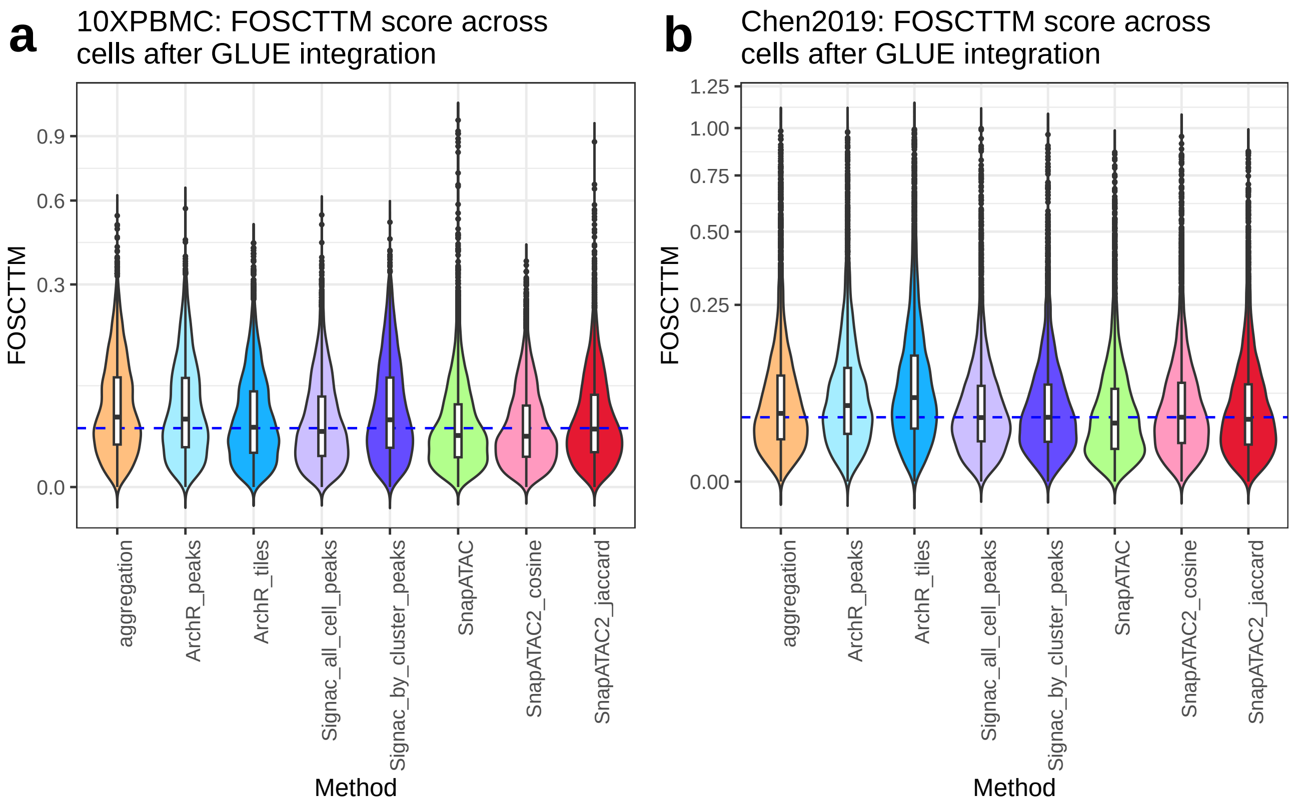

Figure S16: FOSCTTM score in dataset 10XPBMC (a) and Chen2019 (b). The y-axes use a square root scale, and the blue dashed lines represent the median values calculated across the median of each method.

Fig S17

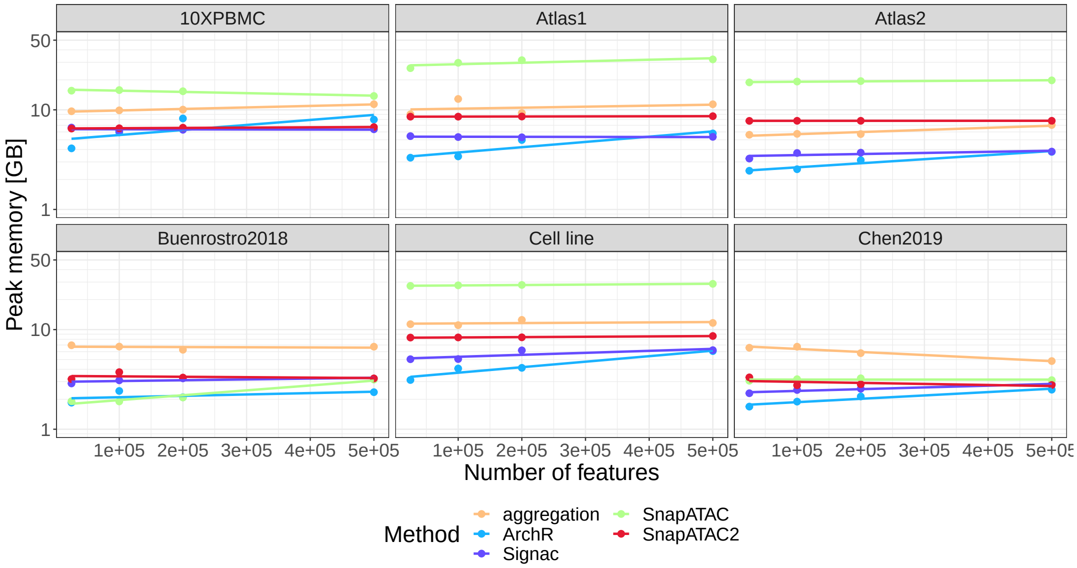

Figure S17: Peak memory usage when using different feature numbers.

Fig S18

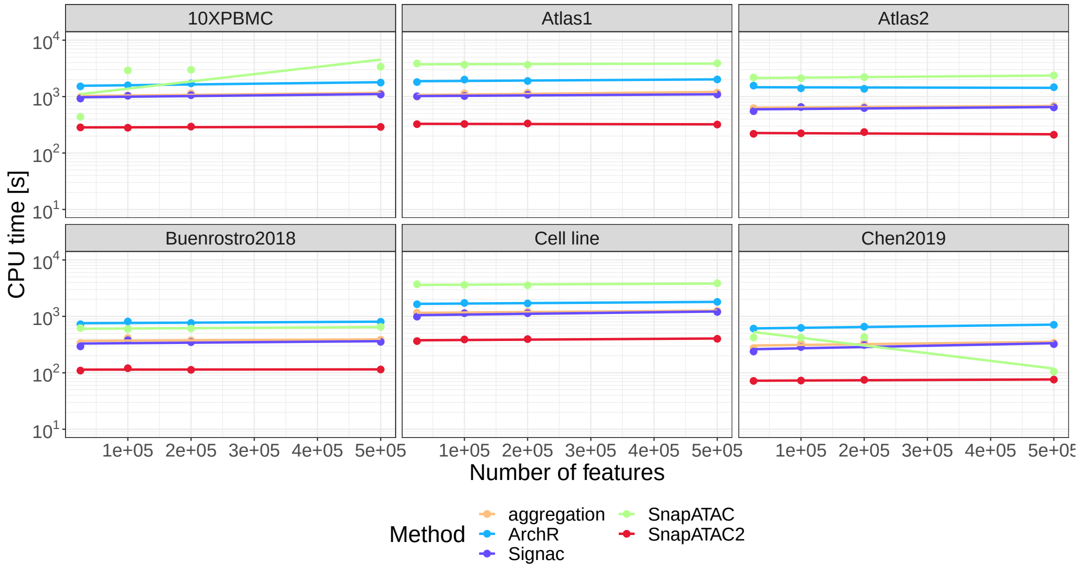

Figure S18: CPU time when using different feature numbers.

Fig S19

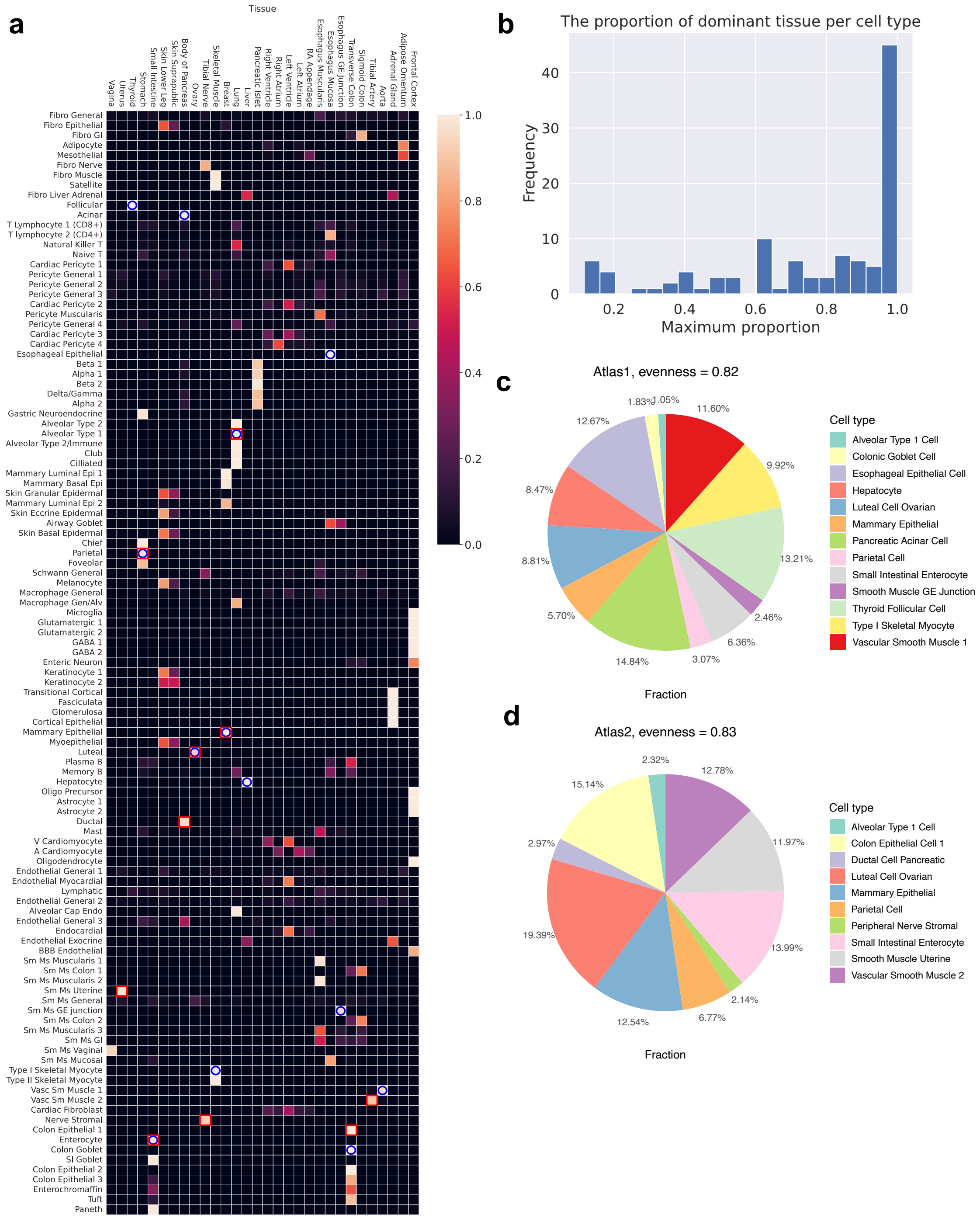

Figure S19: **a** Tissues and cell types in the human adult scATAC-seq atlas, with colors corresponding to column-wise proportion. Blue circles highlight the selected cell classes in Atlas1, and red rectangles highlight cell types in Atlas2. **b** Histogram of the proportion of dominant tissue for each cell type. For most cell types, the most dominant tissue contributes more than 85% cells. **c,d** Cell types and the proportion of our datasets Atlas1, Atlas2. Cell type evenness is calculated. For the evenness of other datasets, see Figure S21.

Fig S20

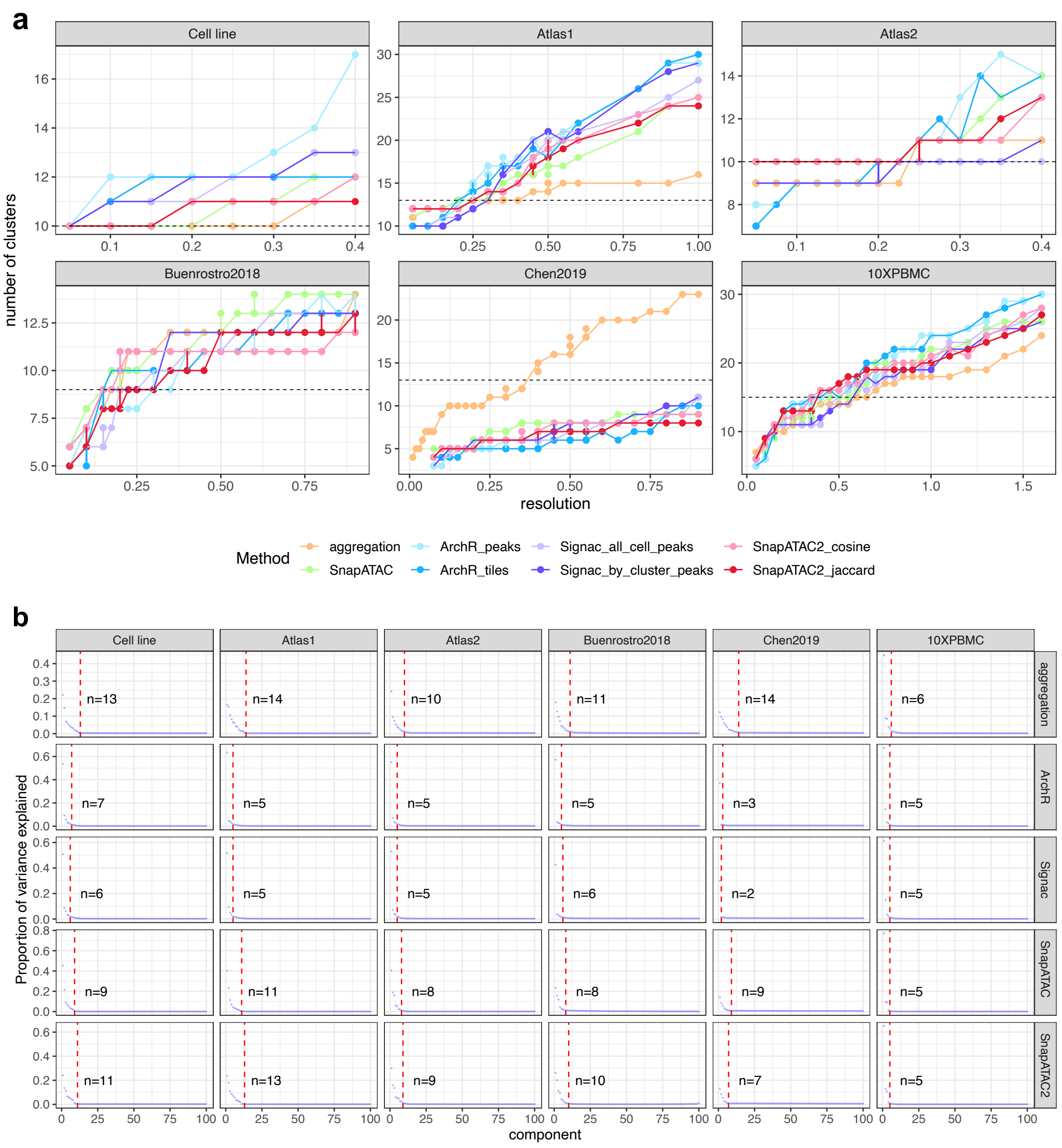

Figure S20: **a** The range of resolutions searched for each dataset, and how the number of clusters change as resolution changes. **b** Elbow plots for each method and datasets.

Fig S21

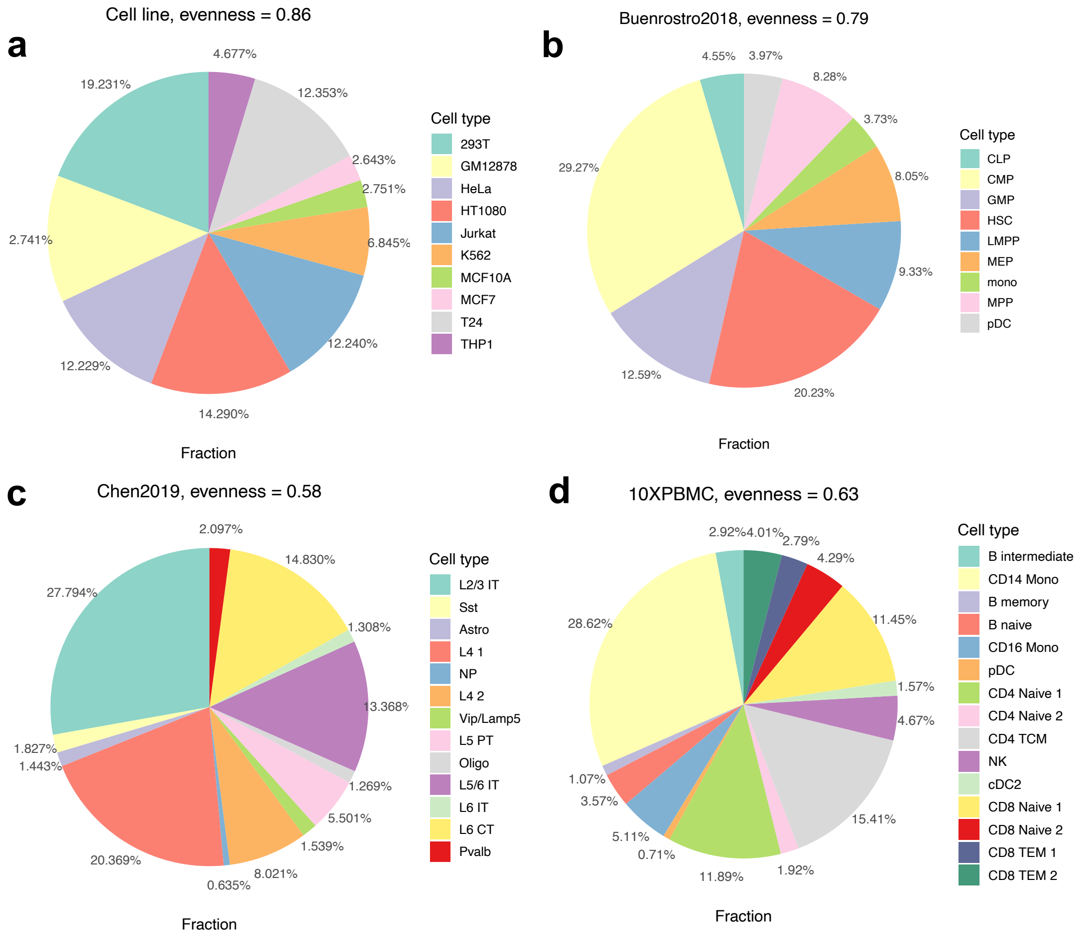

Figure S21: Cell type evenness of other datasets.
