# Supplementary material for: Benchmarking computational methods for single-cell chromatin data analysis
Source: Genome Biol. 2024 Aug 16;25:225. doi: 10.1186/s13059-024-03356-x (PMC11328424; doi:10.1186/s13059-024-03356-x)
Supplement: Supplementary file 3 — Additional file 3. Review history [file 13059_2024_3356_MOESM3_ESM.docx]

**1^st^ round**

**Reviewer 1**

The manuscript has provided a fair and comprehensive comparison of the most popular methods for analyzing scATAC-seq data, especially in terms of their abilities to discern cell states. Overall, the manuscript is clearly-written and benchmarking methods and evaluation metrics make sense.

There are two shortcomings of the current draft that I hope the authors could address in a careful revision:

1. It would be helpful if the authors could compare on the two datasets with joint profiling of RNA and ATAC how different ATAC processing methods facilitate joint analysis with the transcriptome. For instance, the ability to discern cell states when using information from both sides, with the pre-processing of transcriptome data fixed and only varying ATAC processing methods.

2. The analysis of scATAC-seq data is often not used for discerning cell states only. For example, it would be helpful to compare the impact of the different pipelines on predicting gene activities.

**Reviewer 2**

In this manuscript, Luo et al. proposed a benchmarking workflow named Snakemake for assessing computational methods for single-cell chromatin data analysis. The authors evaluated the ability of 8 feature engineering pipelines, derived from 5 recent methods, to discover and discriminate cell types using Leiden clustering. A comprehensive evaluation of the methods and clustering parameter choices was performed at cell embedding, shared nearest neighbor graph, and partition levels. The authors also ranked the methods on datasets with varying degrees of complexity and compared their scalability. While I appreciate its many potential merits, it would be better if the following concerns could be addressed.

Major

1. The line graphs in Figure S5 and S6 may not be appropriate since the horizontal axis represents the datasets, and these two figures do not aim to illustrate the trends of different methods on the datasets.

2. On page 8, the second paragraph of the section "Stability of clusterings and robustness of clustering performance" mentions focusing on the resolution that produces the highest clustering performance. What is the value of this resolution? Does each method have a different optimal resolution for different datasets? The caption of Fig. S11(a) says "x-axis is the deviation of ARI from 1, averaged across resolutions and random seeds." However, different resolutions may result in significantly different numbers of clusters. What is the rationale or basis for averaging the results across different resolutions? The authors need to provide more clear explanations. In addition, the authors are advised to describe how the pairwise ARI is calculated.

3. As shown in Figures S5 and S6, the results obtained by using Jaccard or cosine similarity to construct the affinity matrix are very similar across nine metrics. What are the differences between the clusters obtained by these two methods? The authors could explain in detail the differences between these two construction methods, and the reasons for their similar results.

4. The authors need to provide a clear definition or calculation formula for the library size. How is Pearson's correlation coefficient calculated between the latent dimension and the square root of the library size per cell type?

5. In the section "LSI-based methods show strong library size biases", the authors claimed that LSI-based methods such as Signac and ArchR generate latent representations that are strongly associated with library size, while SnapATAC and SnapATAC2 using Jaccard distance are less affected. In the Signac tutorial, the low-dimensional representation used is from the second to the thirtieth dimension, which may have already eliminated the problem of the first component being affected by sequencing depth. Does the iterative LSI approach used by ArchR involve similar operations? After excluding the first principal component, will the latent representations produced by LSI-based methods still be affected by library size? What are the reasons for the authors' opinion that LSI-based methods such as Signac and ArchR generate latent representations that are strongly associated with library size, while SnapATAC and SnapATAC2 using Jaccard distance are less affected?

6. How many features did each of the compared methods obtain on each dataset? How did the number of features affect the clustering results, time, and peak memory usage of different methods? Were there significant differences in the features selected by different methods?

7. Why did the RNA modality of the 10XPBMC and Chen2019 datasets use different resolutions when performing Leiden clustering in Seurat? What was the reason for using resolution=0.5 for Chen2019 and resolution=0.8 for 10XPBMC? I think the benchmarking could be more fairly.

8. In the section of scRNA-seq data annotation, "For each Leiden cluster, the majority cell label was token as the label of that cluster." Since the cell labels were already transferred from the reference dataset, why did the authors use the Leiden clustering algorithm to re-annotate the cell types? What were the motivation and relevant grounds for using the Leiden clustering algorithm to re-annotate the cell types?

9. What does "the clustering in intermediate steps with increasing resolutions" mentioned on page 14, line 56 refer to?

10. Why did the authors run the function "runDiffusionMaps()" using all cells instead of using landmark cells in SnapATAC? What impact would it have on clustering results, time, and peak memory usage if they strictly followed the SnapATAC tutorial's recommendation to randomly sample a certain number of cells as landmarks?

11. The authors mentioned that LISI calculates the expected number of cells required to be sampled before two cells are drawn from the same batch/clusters within its neighborhood. How is this calculation performed? Should "N" on page 16, line 33 be replaced with "K"?

12. How does ARI2 alleviate the class size bias of ARI?

13. Figure 2 shows the embedding- and graph-level metrics averaged across all cells or all cell classes for each dataset. Averaging may mask some information. What if the authors use box plots instead?

14. The authors reasoned that iterative LSI might contribute more to stabilizing feature selection rather than significantly improving the discernment of difficult-to-distinguish cell types. Then, what is the purpose of stabilizing feature selection?

15. When computing cLISI, how to compute the expected number of cells needed to be sampled before two cells are drawn from the same batch/clusters within its neighborhood for each node in a kNN graph?

Minor

16. It is recommended that the full names of the following abbreviations: ARI, cLISI, PWC score, ARI2, AV, MI, and AW should be provided before their first usage on page 4.

17. For clarity in reading, the author should specify the chapter or section where detailed explanations for each comparative method can be found when it is first introduced.

18. On page 23, in the caption of Figure 3, "c" in the fourth line should be changed to "b".

19. The authors introduced the Evenness (E) metric that can reflect the balance degree of the dataset. Could the authors provide the E values for the datasets they used?

**Authors’ response**

Reviewer 1

It would be helpful if the authors could compare on the two datasets with joint profiling of RNA and ATAC how different ATAC processing methods facilitate joint analysis with the transcriptome. For instance, the ability to discern cell states when using information from both sides, with the pre-processing of transcriptome data fixed and only varying ATAC processing methods.

We thank the reviewer for this intriguing comment. We agree that a comparative study of ATAC processing methods for multi-modal integration would offer valuable insights to the community. However, when considering the feasibility of such an analysis, we have identified some challenges that complicate conducting this investigation thoroughly and arriving at definitive conclusions.

Given that RNA and ATAC data often show consistency in reflecting cell states, it's plausible that improved ATAC processing could enhance the quality of joint analyses. This is because incorporating more modalities can help in reducing noise. However, in our study, the RNA modality is used as the ground truth for benchmarking, primarily due to its technical advantages in terms of providing more fine-grained information and the relative ease of analysis compared to DNA accessibility. If we aim at investigating the integration of both modalities, the RNA data would not be able to serve as an independent ground truth. The cell type annotations from the original papers of the datasets cannot be used for such purpose because those annotations were either also generated from the RNA modality alone, or involved integrated clustering where a specific ATAC-seq processing pipeline was used, indicating that we would not be able to compare the ATAC-seq processing methods fairly. Overall, it is difficult to have an independent ground truth for cell states, and this limits our ability to confirm our hypothesis.

The analysis of scATAC-seq data is often not used for discerning cell states only. For example, it would be helpful to compare the impact of the different pipelines on predicting gene activities.

Thank you for your valuable suggestion. We believe it has led to an interesting addition in our revised manuscript.

Methods for predicting gene activities typically involve aggregating counts from different regions and normalizing their counts. For this reason, it is largely independent of the processing methods investigated in our study. However, we agree with the reviewer that comparing different gene scoring methods from the packages we reviewed would be very informative for users when they are selecting the software.

Although Granja et al. (ArchR manuscript; Nat Gen 2021) already conducted a comprehensive evaluation of these gene activity prediction methods, we performed an independent comparison to validate their findings. This information is now presented in subsection “Benchmarking methods for predicting gene activity scores” in the Results. Interestingly, our findings differ from those of Granja et al.; while their study suggested that ArchR's best model consistently surpasses others like Signac and SnapATAC, we found that ArchR tends to underperform compared to Signac, SnapATAC, and SnapATAC2 (Figure 6 a, c-h). We consider this observation crucial for users to be aware of and believe it underscores the significance of neutral and independent benchmarking of computational tools.

Reviewer 2

The line graphs in Figure S5 and S6 may not be appropriate since the horizontal axis represents the datasets, and these two figures do not aim to illustrate the trends of different methods on the datasets.

Thank you for this note. To avoid any confusion, we now used dot plots in Figure S5 and S6 in the revised manuscript.

On page 8, the second paragraph of the section "Stability of clusterings and robustness of clustering performance" mentions focusing on the resolution that produces the highest clustering performance. What is the value of this resolution? Does each method have a different optimal resolution for different datasets?

Yes, as the reviewer pointed out, the resolution value of the highest ARI is different mainly across datasets, but also across methods. We now put the optimal resolution values in “Additional file 1” for the reader's reference.

The caption of Fig. S11(a) says "x-axis is the deviation of ARI from 1, averaged across resolutions and random seeds." However, different resolutions may result in significantly different numbers of clusters. What is the rationale or basis for averaging the results across different resolutions? The authors need to provide more clear explanations. In addition, the authors are advised to describe how the pairwise ARI is calculated.

We thank the reviewer for pointing out this confusion and we have expanded the explanation within the "Analysis of clustering robustness" subsection (see Methods) to clarify this point. Basically, clustering stability is distinct from accuracy. It examines the consistency of a methods’ results across multiple executions. Pairwise ARI is used to compare how similar the clustering results are across random seeds when fixing the resolution. An ARI of 1 indicates identical clusterings, whereas an ARI of 0 means that the two clusterings are independent. We calculated the deviation of pairwise ARI from 1, interpreting this as a measure of the clustering's robustness when fixing resolutions. When averaging this deviation across resolutions, it indicates an overall estimation of clustering robustness of a method. Therefore, the number of clusters generated does not impact this analysis.

As shown in Figures S5 and S6, the results obtained by using Jaccard or cosine similarity to construct the affinity matrix are very similar across nine metrics. What are the differences between the clusters obtained by these two methods? The authors could explain in detail the differences between these two construction methods, and the reasons for their similar results.

SnapATAC2 first constructs a cell-to-cell similarity matrix and then calculates the spectral embeddings for these cells. The major differences between the two construction methods discussed include: (i) the similarity metrics applied in the construction of the similarity matrix; and, (ii) the algorithms implemented for the calculation of the spectral embeddings.

Specifically, when applying cosine similarity, eigenvectors are derived using the Lanczos algorithm, which eliminates the need for constructing a full similarity matrix. This alteration affects only the computational efficiency and does not change the outcomes compared to using a full matrix.

Our analysis reveals that the clusters obtained by using either Jaccard or cosine similarity are very similar across our datasets, as shown in Reviewer Figure 1. However, identifying the exact reason behind this similarity is challenging. We considered evaluating the similarity matrices created through Jaccard and cosine similarity. However, since the implementation of dimensional reduction in SnapATAC2 is wrapped in a single function “snapatac2.tl.spectral()”, we found it hard to deconvolute the individual step of matrix construction from this function.

Reviewer Figure 1: Confusion matrix of the clustering results inferred by using cosine similarity and Jaccard similarity in SnapATAC2. The clustering parameters were set to be the ones that give the highest ARI for each method

The authors need to provide a clear definition or calculation formula for the library size. How is Pearson's correlation coefficient calculated between the latent dimension and the square root of the library size per cell type?

To avoid any confusion, we have now expanded the explanation in the “Analysis of library size biases” subsection (see Methods). We defined the library size of each cell as the total number of unique fragments of that cell in the fragment file and used formula 15 (page 22) to explain the calculation of Pearson’s correlation coefficient.

In the section "LSI-based methods show strong library size biases", the authors claimed that LSI-based methods such as Signac and ArchR generate latent representations that are strongly associated with library size, while SnapATAC and SnapATAC2 using Jaccard distance are less affected. In the Signac tutorial, the low-dimensional representation used is from the second to the thirtieth dimension, which may have already eliminated the problem of the first component being affected by sequencing depth. Does the iterative LSI approach used by ArchR involve similar operations? After excluding the first principal component, will the latent representations produced by LSI-based methods still be affected by library size?

As the reviewer pointed out and as we mentioned in the Methods section, in Signac’s tutorial, a filtering step of removing latent components that are highly correlated with the library counts is recommended. In our study, this filtering was applied to all methods. Notably, this procedure consistently led to the removal of the first components in both Signac and ArchR's outputs, whereas it did not affect any components in the other methods examined. Our analysis on library size biases indicates that the latent representations generated by LSI-based methods remain significantly influenced by library size. Despite following the filtering procedure recommended by the authors of Signac, this issue persists.

What are the reasons for the authors' opinion that LSI-based methods such as Signac and ArchR generate latent representations that are strongly associated with library size, while SnapATAC and SnapATAC2 using Jaccard distance are less affected?

We thank the reviewer for raising this question. While we are unable to definitively determine the mechanism responsible for this observation, we are happy to share our opinions on this. With sparse matrices, Jaccard distance can be very sensitive to library size (Fang, Rongxin, et al., Nature communications 2021). SnapATAC specifically regresses this effect out to normalize the observed Jaccard similarity by modelling the relationship between observed and expected Jaccard similarity given the library sizes. This can be the reason that SnapATAC is less affected by library size. In LSI-based methods, instead of the absolute frequency, the relative frequency of a term (feature) in a document (cell) is calculated as the term frequency (TF), which can be interpreted as a way to mitigate the library size effects as well. However, according to our results, residual effects still remain in the output of LSI-based methods. In our opinion, a potential reason is that the reads from scATAC-seq are not fully quantitative.

How many features did each of the compared methods obtain on each dataset? How did the number of features affect the clustering results, time, and peak memory usage of different methods? Were there significant differences in the features selected by different methods?

We thank the reviewer for this comment. Indeed, the compared methods use different approaches for feature selection, and by default select different numbers of features for dimensional reduction. ArchR selected the most variable features across clusters using an iterative approach, and the top 25k features are retained. Signac selected the top 95% most- accessible features, meaning it’s around 200k peaks. SnapATAC filtered out the top 5% most accessible (and thus least variable) bins, while SnapATAC2 performed both the upper and lower quantile filtering. Both SnapATAC and SnapATAC2 use around 500k bins. We performed an analysis by running each method using a grid of feature numbers, including 25k, 100k, 200k, and 500k. We observed that as the number of features changes, the clustering performance are relatively stable, although ArchR appears to benefit from slightly higher number of features than used by default, and snapATAC did not perform well with lower number of features. The memory and time usage is stable as well, except that ArchR has a slightly increasing peak memory usage as the feature number increases. This analysis and the detailed result Figures are now added to the revised manuscript as supplementary notes in “Additional file 2”.

Why did the RNA modality of the 10XPBMC and Chen2019 datasets use different resolutions when performing Leiden clustering in Seurat? What was the reason for using resolution=0.5 for Chen2019 and resolution=0.8 for 10XPBMC? I think the benchmarking could be more fairly.

Thank you for your comments. The resolutions used for clustering the RNA modality of the 10XPBMC and Chen2019 datasets are determined independently of the benchmark, and using the same procedure. For each dataset, we first performed Leiden clustering using a series of resolution values (with the maximum resolution obviously over-clustering the datasets), and constructed a cluster tree showing the co-clustering consistency across resolutions (Zappia and Oshlack, GigaScience 2018). Then we chose a resolution value that gives stable clustering (in the sense that increasing resolution does not lead to cells that were split at a higher resolution to be placed back in the same cluster) and reasonable separation of cells in the UMAP for each dataset. Our reasoning here was that RNA separation ought to be robust and unambiguous if it is to serve as ground truth for the ATAC modality. Naturally, the most stable resolution is not the same for the two datasets; nevertheless, we consider the benchmark to be entirely fair because the procedure is a fairly standard one, has been applied similarly to both datasets, and the final resolution was selected independently of the ATAC results. To avoid any confusion, we have now expanded the explanation in the method section in the revised manuscript.

In the section of scRNA-seq data annotation, "For each Leiden cluster, the majority cell label was token as the label of that cluster." Since the cell labels were already transferred from the reference dataset, why did the authors use the Leiden clustering algorithm to re-annotate the cell types? What were the motivation and relevant grounds for using the Leiden clustering algorithm to re-annotate the cell types?

Label transfer is known to be imperfect. The transferred labels can be noisy, and the reference can have significant differences to the dataset at hand. As the benchmark depends more on consistency between modalities in the dataset, and less on the generalizable biological identities of the cells, it does not make sense if two cells known to be very similar in the embedding space get two different labels. Therefore, we performed clustering using the RNA modality and used the RNA clusters as our ground truth for ATAC clusters. Label transfer and the following majority voting is a strategy for labelling the RNA clusters.

What does "the clustering in intermediate steps with increasing resolutions" mentioned on page 14, line 56 refer to?

For instance, when you execute addIterativeLSI() and set clusterParams to list(resolution = c(0.1, 0.2, 0.4)) in ArchR, it initiates four iterations of LSI accompanied by clustering three times. Each clustering step uses a different resolution – specifically 0.1, 0.2, and 0.4, in that order. This approach is suggested in ArchR's documentation as an effective method for better selecting highly variable features across different clusters.

Why did the authors run the function "runDiffusionMaps()" using all cells instead of using landmark cells in SnapATAC? What impact would it have on clustering results, time, and peak memory usage if they strictly followed the SnapATAC tutorial's recommendation to randomly sample a certain number of cells as landmarks?

We thank the reviewer for raising this question. We consistently used the entire dataset rather than selecting a subset of cells in SnapATAC, SnapATAC2, and ArchR, which all have some sort of subsampling procedure to accelerate the computation, to ensure a fair comparison with other methods. Given that the datasets employed are of relatively small or moderate size, running all methods using all cells doesn't pose significant efficiency issues, which is the primary concern that subsampling procedures are designed to address. To avoid any confusion, we have now stated this in the Methods section in the revised manuscript. To assess the effects of the subsampling approach recommended in the SnapATAC tutorial on clustering efficacy, processing time and memory consumption, we implemented a benchmark analysis. This involved using subsets of 50%, 80%, and 100% of cells as landmark cells within the "runDiffusionMaps()" function. The outcomes, evaluated using metrics such as ARI, ARI2, and MI, are depicted in Reviewer Figure 2. The findings indicate that subsampling did not compromise the clustering results. On the other hand, as illustrated in Reviewer Figure 3, while the reduction in the number of landmark cells did not decrease CPU time, it notably decreased peak memory usage.

Reviewer Figure 2: The clustering performance of SnapATAC, measured by ARI (A), ARI2 (B) and MI (C), using 50%, 80%, and 100% of cells as landmark cells in the function "runDiffusionMaps()".

Reviewer Figure 3: CPU time (a) and peak memory usage (b) of SnapATAC when using 50%, 80%, and 100% of cells as landmark cells in the function "runDiffusionMaps()".

The authors mentioned that LISI calculates the expected number of cells required to be sampled before two cells are drawn from the same batch/clusters within its neighborhood. How is this calculation performed?

The calculation of LISI is implemented by using the function compute_lisi() from the R package lisi v1.0. We have added the details in the “Methods” section.

Should "N" on page 16, line 33 be replaced with "K"?

Yes, thank you for pointing it out. We have now corrected it in the revised manuscript.

How does ARI2 alleviate the class size bias of ARI?

Equation (4)-(7) indicate that ARI is dependent on the cluster sizes (reflected by P_i and Q_j), while ARI2 is invariant to cluster size.

Figure 2 shows the embedding- and graph-level metrics averaged across all cells or all cell classes for each dataset. Averaging may mask some information. What if the authors use box plots instead?

Thank you for your suggestion. We agree that averaging across all cell classes can mask some information. We tried to visualize using box plots (see Reviewer Figure 4) and realized that the distribution across cell classes can have large spread and making it hard to read and compare between methods. We argue that in this case the “outlier” cell classes should not be excluded visually from comparison, and therefore the boxplot is less intuitive for visualization. Thus, we decided to present the data using barplots as we did before.

Reviewer Figure 4: Boxplot showing the proportion of weakly connected cells (PWC) across cell types.

The authors reasoned that iterative LSI might contribute more to stabilizing feature selection rather than significantly improving the discernment of difficult-to-distinguish cell types. Then, what is the purpose of stabilizing feature selection?

ArchR’s author claimed that by selecting the most variable features across clusters instead of using the most accessible features, the reproducibility between samples can be improved (Satpathy, Granja et al. Nature Biotechnology 2019 and Granja, Klemm and McGinnis et al. Nature Biotechnology 2019). We thought that this is because the most variable features across clusters can be more robust to technical effects compared to the most accessible features. To make it clear, we rephrase this discussion in the manuscript.

When computing cLISI, how to compute the expected number of cells needed to be sampled before two cells are drawn from the same batch/clusters within its neighborhood for each node in a kNN graph?

As our response to comment 11 above, the calculation of LISI is implemented by using the function compute_lisi() from the R package lisi v1.0. We have added the details in the “Methods” section.

Reviewer Figure 4: Boxplot showing the proportion of weakly connected cells (PWC) across cell types.

The authors reasoned that iterative LSI might contribute more to stabilizing feature selection rather than significantly improving the discernment of difficult-to-distinguish cell types. Then, what is the purpose of stabilizing feature selection?

ArchR’s author claimed that by selecting the most variable features across clusters instead of using the most accessible features, the reproducibility between samples can be improved (Satpathy, Granja et al. Nature Biotechnology 2019 and Granja, Klemm and McGinnis et al. Nature Biotechnology 2019). We thought that this is because the most variable features across clusters can be more robust to technical effects compared to the most accessible features. To make it clear, we rephrase this discussion in the manuscript.

When computing cLISI, how to compute the expected number of cells needed to be sampled before two cells are drawn from the same batch/clusters within its neighborhood for each node in a kNN graph?

As our response to comment 11 above, the calculation of LISI is implemented by using the function compute_lisi() from the R package lisi v1.0. We have added the details in the “Methods” section.

It is recommended that the full names of the following abbreviations: ARI, cLISI, PWC score, ARI2, AV, MI, and AW should be provided before their first usage on page 4.

Thank you for your suggestion. We have taken this advice in the revised manuscript and added the full names.

For clarity in reading, the author should specify the chapter or section where detailed explanations for each comparative method can be found when it is first introduced.

Thank you for highlighting this issue. We have addressed it in the revised manuscript.

On page 23, in the caption of Figure 3, "c" in the fourth line should be changed to "b".

Thank you for highlighting this issue. We have corrected it in the revised manuscript and figures.

The authors introduced the Evenness (E) metric that can reflect the balance degree of the dataset.

Could the authors provide the E values for the datasets they used?

The E values for other datasets are now shown in Figure S16.

**2^nd^ round**

**Reviewer 1**

The authors have successfully addressed my second concern in the first round report while arguing that it is not possible to address my first comment. This is disappointing. There are multiple ways of comparing how pre-processing of the ATAC assay affects integrative analysis of multiome measurements of single cells.

For example, one could fix the pre-processing of the RNA assay, use different pre-processing pipelines for ATAC, and fix the integration pipeline of the pre-processed assays. Thus, the only factor in the pipeline is the ATAC pre-processing method. Metrics, such as the average Silhoutte score and the network modularity applied on Leiden clustering labels, could then be used to quantitatively evaluate the quality of the resulting multiome single-cell atlases. These metrics do not depend on a user-defined set of cell type annotations, hence avoiding the main concern the authors put in the rebuttal letter for not being able to address my first comment. Given its feasibility, I believe the authors could provide benchmarking results along this line in a further revision.

**Reviewer 2**

All the issues have been addressed.

**Authors’ response**

*Reviewer #1: The authors have successfully addressed my second concern in the first round report while arguing that it is not possible to address my first comment. This is disappointing. There are multiple ways of comparing how pre-processing of the ATAC assay affects integrative analysis of multiome measurements of single cells.*

*For example, one could fix the pre-processing of the RNA assay, use different pre-processing pipelines for ATAC, and fix the integration pipeline of the pre-processed assays. Thus, the only factor in the pipeline is the ATAC pre-processing method. Metrics, such as the average Silhoutte score and the network modularity applied on Leiden clustering labels, could then be used to quantitatively evaluate the quality of the resulting multiome single-cell atlases. These metrics do not depend on a user-defined set of cell type annotations, hence avoiding the main concern the authors put in the rebuttal letter for not being able to address my first comment. Given its feasibility, I believe the authors could provide benchmarking results along this line in a further revision.*

We understand the request of the reviewer and appreciate the suggestion to use Leiden clustering outcomes from integration analysis for evaluation, which tries to address our earlier concerns about the feasibility and interpretability of benchmarking integration analysis without adequate ground truth. We nonetheless still have some reservations about the interpretation of the results that would come from the analysis suggested since using the clusters obtained from the integration for evaluation poses some risks. First, since each method generates its own clusters and embedding, the metrics are not entirely comparable across methods. Second, it is very plausible for a method to produce false clusters that are well separated in its embedding or graph. This is especially true in the context of scATAC-seq, given the difficulties we have shown to adequately normalise differences in library sizes. It is further confounded by some components of the methods evaluated, such as ArchR's iterative feature selection, which can produce clusters that are artificially over-segregated.

This being said, we did feel that there is a legitimate interest in the reviewer’s request, and we therefore tried to achieve the same aims through slightly different means. As the reviewer suggested, we kept the RNA and integration methods fixed, but instead of performing paired integration, we treated the cells from the two modalities as if they were unmatched (that is, unpaired integration), and used the pairing information as the ground truth for evaluation. The rationale behind this approach is that if an ATAC preprocessing method is effective in facilitating robust integration with RNA, then the corresponding matched cells should be close to each other in the integrated space. This analysis also has the advantage of not relying on the RNA clusters. We have added this analysis in the Results section “Differences

in scATAC-seq processing propagate to integration with scRNA-seq” (see especially (Supplementary Figure 21). While the results are comparable across methods, the general trends were consistent with our previous benchmarks (e.g. good SnapATAC1/2 performance, poorer ArchR & aggregation performance, and Signac somewhere in-between), further strengthening the overall picture.

Using paired integration and evaluating on the RNA clusters (as in the main benchmark) yielded a similar, though blurrier picture (Figure R1 below). Given the similar conclusions and the caveats of the paired-integration approach, we therefore chose to include the above-mentioned unpaired approach in the manuscript.

Figure R1: Metric values when comparing clustering results of WNN integration to that of ATAC only (evaluated against the RNA clusters, as in the main benchmark). The y-axis of PWC is in square root scale. As expected, using RNA+ATAC better recapitulates the RNA clusters.

**3^rd^ round**

**Reviewer 1**

The authors have successfully addressed all my concerns
